# Association Between Foot Morphology and Hallux Valgus Angle During Rotational Motion

**DOI:** 10.7759/cureus.87859

**Published:** 2025-07-13

**Authors:** Toshihiko Sato

**Affiliations:** 1 Department of Physical Therapy, Bunkyo Gakuin University, Fujimino, JPN

**Keywords:** foot mobility, foot shape, hallux angle, hallux valgus, rotational motion

## Abstract

Background:The foot changes shape in response to rotational motion; however, the specific nature of these changes and their relationship to hallux valgus remain unclear. In this study, we aimed to examine the relationship between foot shape alterations due to rotational motion and the hallux valgus angle.

Methods:Foot shape in healthy adult participants was measured using a three-dimensional (3D) foot scanner. Differences in foot morphology due to rotational motion were analyzed using Dunnett’s multiple comparisons method. Additionally, multiple regression analysis was conducted to identify foot shape factors associated with changes in the hallux angle.

Results:On the rotational side, the foot arch was higher, and the hallux angle tended toward varus. In contrast, on the non-rotational side, the arch was lower, and the hallux angle tended toward valgus. The forefoot, midfoot, and medial-lateral malleolus inclination angles showed angular patterns that opposed changes in the calcaneus inclination angle. Multiple regression analysis revealed that, on the rotational side, higher forefoot height and varus displacement of the digitus minimus were associated with a more varus displacement of the hallux angle. On the non-rotational side, a lower navicular height, valgus displacement of the digitus minimus, lower forefoot height, and an everted midfoot inclination angle were associated with a more valgus displacement of the hallux angle.

Conclusion:Foot shape changes induced by rotational motion are influenced by the kinetic chain and weight-bearing position resulting from lower leg rotation. The hallux valgus angle appears to be modulated by both forefoot transverse height and medial longitudinal arch function. These findings may inform targeted interventions such as exercise therapy and custom insole design.

Clinical relevance: This study suggests that insoles providing support to the forefoot transverse and medial longitudinal arches may help mitigate hallux valgus progression by reducing mechanical stress on the hallux.

## Introduction

Hallux valgus is a disorder characterized by lateral deviation of the hallux and medial deviation of the first metatarsal, resulting in pain [1], gait disability, falls [2], and other foot disorders [3] as the condition progresses. The condition is more prevalent among older women [4], with contributing factors including hereditary traits [5], such as genetic predisposition, and the influence of footwear [6]. However, asymptomatic individuals are less likely to seek medical care, suggesting that the actual prevalence may be underreported.

Treatment options for hallux valgus include conservative therapies, such as exercise and orthotic use [7], as well as surgical interventions [8]. However, no definitive treatment has been established. While surgical correction can improve the hallux valgus angle, recurrence has been reported in some cases, particularly depending on the surgical technique and postoperative conditions [3]. Therefore, it is essential to evaluate not only the hallux valgus angle but also the overall foot shape to determine appropriate treatment methods.

Conventional evaluation methods for hallux valgus include X-rays [9], footprints [10], and foot shape analysis [11], all of which typically use the hallux valgus angle as an index. Most evaluation methods focus on isolated regions rather than assessing the entire foot holistically. Since 2000, a technique that segments the foot into multiple parts to measure movement has been introduced [12]; however, this technique is susceptible to error due to skin motion artifacts [13]. In addition, quantitatively capturing the three-dimensional (3D) foot changes during gait evaluation is difficult because of substantial variations in load magnitude and distribution. The navicular bone, although considered the most mobile bone in the foot, moves only approximately 5 mm of under load [14], necessitating palpation-based confirmation of bony landmarks and the use of smaller measurement markers for accuracy.

The foot changes shape three-dimensionally [15] owing to kinetic chain and muscle activity [16] associated with body weight distribution [11] and lower leg rotation [17]. This foot mobility is also thought to reduce mechanical stress on the knee and hip joints during gait [18], and a similar effect may apply to valgus stress on the hallux [19]. Additionally, the rotational motion commonly involved in sports and daily activities [12] likely generates as much valgus stress on the hallux [20] as that produced during normal gait. Understanding the relationship between hallux angle changes and foot hypermobility or hypomobility during rotational motion may contribute to controlling and improving the progression of hallux valgus through appropriate exercise therapy and the development of supportive footwear and insoles.

In this study, we aimed to analyze foot shape changes in response to rotational motion under loading conditions in young individuals without hallux valgus and to clarify the relationship between foot mobility and the hallux valgus angle. We hypothesized that during rotational motion, the foot on the rotational side, which supports body weight, tends to adopt an inverted position, elevating the medial arch and resulting in a smaller hallux valgus angle. In contrast, the foot on the non-rotational side may adopt an everted position, flattening the medial arch and resulting in a larger hallux valgus angle. We aimed to identify general morphological trends in foot behavior under rotational stress and determine the factors that influence the progression or mitigation of hallux valgus.

## Materials and methods

Participants

This cross-sectional observational study was conducted between September 20, 2023, and June 10, 2024. Forty-five healthy participants (90 feet) from the participating institution were included, comprising 22 males and 23 females, with a mean age of 21.3 ± 0.6 years, and average height and weight of 165.0 ± 8.5 cm and 59.0 ± 11.2 kg, respectively. Participants volunteered for this study after seeing a recruitment poster at Bunkyo Gakuin University. All participants reported no history of lower-extremity pain or disorders and confirmed their ability to walk unassisted. Written informed consent was obtained from all participants prior to enrollment.

A power analysis was conducted to assess differences in foot shape among three groups based on foot condition and posture: (1) foot in the static standing position, (2) foot on the rotational side during rotational motion, and (3) foot on the non-rotational side during rotational motion. The results indicated that a minimum sample size of 45 participants (90 feet) was required to achieve a power of 0.8, an effect size (f) of 0.25, and a two-sided significance level of 0.05. This analysis was performed using the G*Power 3.1.9.2 analysis program (Düsseldorf, Germany). The study was approved by the Ethics Review Committee of the participating institution (approval no: 2022-0004) and was conducted in accordance with the Declaration of Helsinki. All measurements were performed by a single examiner with over 10 years of experience in each measurement method.

Ankle-foot complex measures

The ankle-foot complex was assessed using a 3D foot scanner (INFOOT USB scanning system, IFU-S-01, I-Ware Laboratory, Osaka, Japan). Landmark stickers (diameter: 5 mm) were used as reference points and placed on anatomical landmarks according to the manufacturer’s guidelines. Participants were instructed to place one foot in the scanner while positioning the opposite foot on a step of equal height next to the scanner’s glass plate.

**Figure 1:**
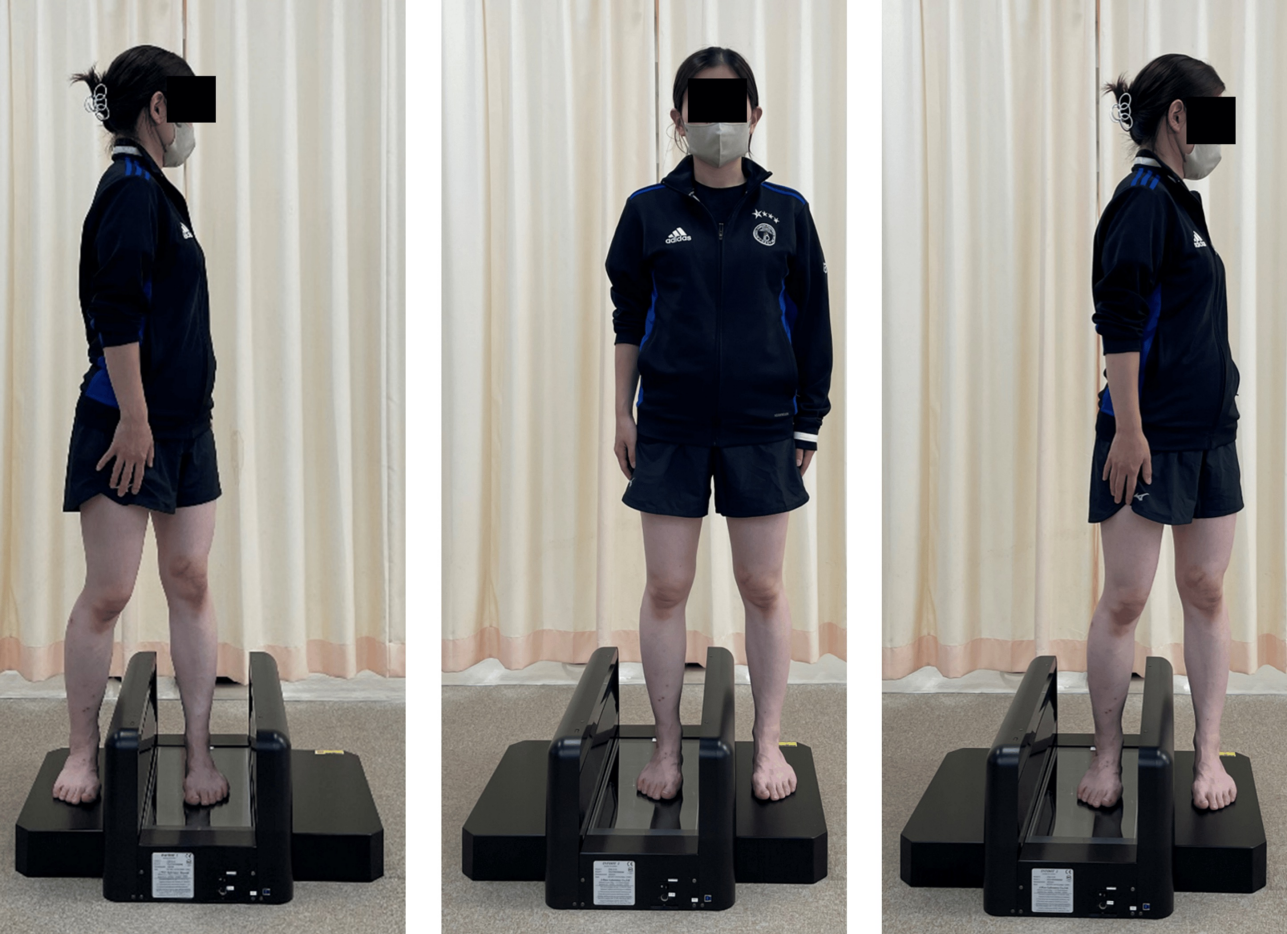
Foot posture during rotational motion measurement. Illustration of the three foot positions analyzed in this study. From left to right: measurement of the foot on the rotational side, the foot in the neutral standing position, and the foot on the non-rotational side. Rotational Side (Left): The measured foot supports the body during rotational movement. It typically demonstrates inversion of the hindfoot and elevation of the medial longitudinal arch. Standing Position (Center): A neutral bilateral stance is used as the baseline reference for all comparisons. Non-Rotational Side (Right): The contralateral foot is measured, which tends to bear less load during rotation. It often shows eversion and medial arch flattening due to anterior weight shift.

For the standing measurement, participants were instructed to remain stationary, relaxed, and distribute their body weight evenly across both feet. For rotational position measurements, participants were instructed to avoid shifting their weight as long as the plantar surface of the foot remained in contact with the scanner. The range of motion was defined as the maximum angle at which the sole of the foot remained in contact with the ground (Figure 1).

The 3D foot scanner enabled multiple measurements to evaluate foot characteristics, including foot length, foot width, heel width, forefoot height, dorsum foot height, navicular height, hallux angle, digitus minimus angle, and calcaneus inclination angle. Forefoot height was normalized to foot width, and dorsum foot and navicular bone heights were normalized to foot length to account for variations in foot size.

**Figure 2:**
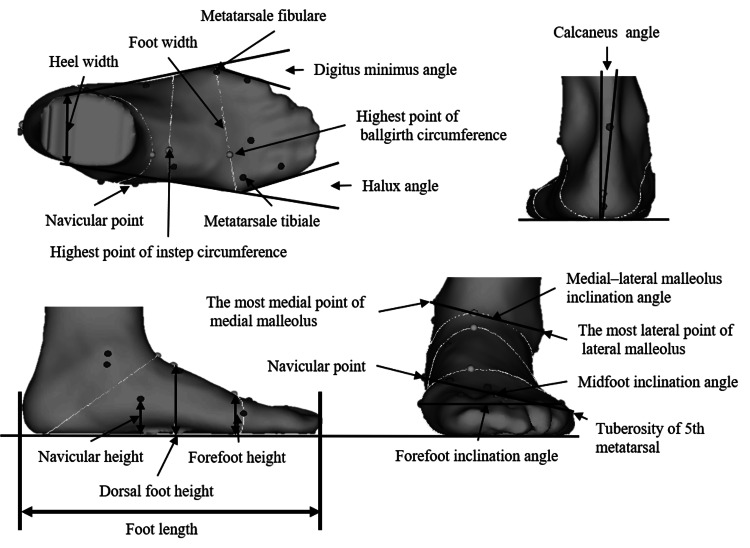
Foot posture measurement indices. Foot length (centimeter [cm]): Longest straight-line distance from the heel point to the midpoint of the foot width. Foot width (cm): Distance between the metatarsal tibial (MT) point and the metatarsal fibular (MF) point. Heel width (cm): Perpendicular distance from the heel point to the foot axis at 16% of total foot length. Forefoot height (cm): Maximum height from the ground measured at the forefoot circumference. Dorsal foot height (cm): Height above the ground at 50% of the total foot length. Navicular height (cm): Vertical distance from the lowest point of the navicular bone to the ground. Hallux angle (in degrees [°]): Angle between the line from the MT point to the end of the hallux and the line from the MT point to the heel width axis; positive for valgus, negative for varus. Digitus minimus angle (°): Angle between the line from the MF point to the fifth toe and the line from the MF point to the heel width axis; positive for varus, negative for valgus. Calcaneus angle (°): Rearfoot angle viewed posteriorly; positive indicates eversion, negative indicates inversion. Forefoot inclination angle (°): Angle of the line connecting the MT and MF points; positive for inversion, negative for eversion. Midfoot inclination angle (°): Angle between the lowest navicular point and the fifth metatarsal bone; positive for inversion, negative for eversion. Medial–lateral malleolus inclination angle (°): Angle between the line connecting the outermost medial and lateral malleoli; positive for inversion, negative for eversion. Note: The first nine indices are based on standard INFOOT definitions [21]. The last three angular indices were originally developed in our previous study [22]. This image is an original creation by the author.

All measurements were converted into a 3D right-hand coordinate system using a file converter (I-Ware Laboratory). In this coordinate system, the heel point was set as the origin: the X-axis represented the foot axis direction, the Y-axis represented foot width, and the Z-axis represented height. The foot arch angle was calculated as the angle between two points on the Y-Z plane relative to the ground. Additional calculated angles included forefoot, midfoot, and medial-lateral malleolus inclination angles. Detailed definitions and measurement procedures for these indices are shown in Figure 2 [21,22].

Statistical analysis

To examine foot morphology and its influence on hallux valgus angle during rotational motion, we conducted the following statistical analyses. First, we assessed the normality of each foot shape variable using the Shapiro-Wilk test. All indices followed a normal distribution. Intra-examiner reliability was assessed using intraclass correlation coefficients, with all indices achieving values ≥0.95, indicating excellent reliability. Second, to evaluate morphological differences across conditions, a repeated-measures one-way analysis of variance was performed to compare foot shape variables among three positions: the reference standing posture, the rotational side, and the non-rotational side during rotational motion. When significant differences were found, Dunnett’s test was applied for multiple comparisons, with the standing position as the reference. Furthermore, we performed direct comparisons of foot shape between the rotational and non-rotational sides under the same standing condition. Third, to identify which morphological parameters contributed to changes in the hallux valgus angle, we calculated the change in each foot shape index as the difference between the rotational or non-rotational side and the reference standing position. Stepwise multiple regression analysis was then conducted, with the change in hallux valgus angle as the dependent variable. The following morphological indices were included as independent variables: forefoot height, dorsum foot height, navicular height, digitus minimus angle, calcaneal inclination angle, forefoot inclination angle, midfoot inclination angle, and medial-lateral malleolus inclination angle. Variance inflation factors (VIFs) were calculated to evaluate multicollinearity among independent variables. All statistical analyses were performed using SPSS (version 26.0; IBM Corp., Armonk, NY, USA), with statistical significance set at P < 0.05.

## Results

Using Dunnett’s method for multiple comparisons, the results showed that forefoot height, dorsum foot height, and navicular height were significantly higher in the rotational side than in the standing position. Furthermore, the hallux and digitus minimus angles exhibited varus displacement, while the forefoot and midfoot inclination angles showed inversion. Conversely, on the non-rotational side, the forefoot, dorsum of the foot, and navicular were positioned lower; the hallux angle showed valgus displacement; and the forefoot, medial-lateral malleolus, and calcaneal inclination angles exhibited eversion (Table 1). On the rotational side, the forefoot and midfoot inclination angles were everted toward the calcaneus, whereas on the non-rotational side, the forefoot, midfoot, and medial-lateral malleolus inclination angles were inverted relative to the calcaneus (Table 2).

**Table 1:** Comparison of foot shape parameters between rotational and non-rotational sides during standing *P < 0.05, **P < 0.01 (vs. standing position, Dunnett’s method). Values are expressed as mean ± standard deviation (SD). Significant differences indicate changes in foot morphology associated with rotational motion relative to standing posture.

Parameter	Non-rotational side (Mean ± SD)	Standing (Mean ± SD)	Rotational side (Mean ± SD)
Forefoot height (％)	35.7 ± 2.3^**^	36.2 ± 2.5	36.8 ± 2.6^**^
Dorsum foot height (％)	24.2 ± 1.9^**^	24.6 ± 1.9	25.2 ± 2.1^**^
Navicular height (％)	12.5 ± 2.4^**^	13.5 ± 2.3	14.3 ± 2.4^**^
Hallux valgus angle (in degrees (º)	12.6 ± 5.1^**^	11.3 ± 5.1	9.2 ± 5.9^**^
Digitus minimus varus angle (º)	10.4 ± 4.3	10.9 ± 4.5	11.8 ± 4.6^*^
Forefoot inclination angle (º )	2.9 ± 1.9^**^	4.6 ± 1.9	6.5 ± 2.0^**^
Midfoot inclination angle (º)	7.6 ± 7.1	9.0 ± 4.6	11.1 ± 4.9^*^
Medial–lateral malleolus inclination angle (º)	10.2 ± 4.0^**^	11.7 ± 3.4	12.0 ± 3.4
Calcaneus eversion angle (º)	3.7 ± 2.4^**^	2.7 ± 2.3	2.9 ± 2.6

**Table 2:** Horizontal plane inclination angles of foot segments relative to the calcaneus *P < 0.05, **P < 0.01 (vs. standing position, Dunnett’s method). Values are expressed as mean ± standard deviation (SD). Positive values indicate eversion relative to the calcaneus; negative values indicate inversion. Significant differences reflect rotational motion effects on transverse foot segment alignment.

Parameters	Non-rotational side (Mean ± SD)	Standing (Mean ± SD)	Rotational side (Mean ± SD)
Forefoot inclination angle (º)	-0.8 ± 3.4**	1.9 ± 3.4	3.6 ± 3.8**
Midfoot inclination angle (º)	3.8 ± 8.2 *	6.3 ± 6.0	8.2 ± 6.4**
Medial–lateral malleolus inclination angle (º)	6.4 ± 5.5**	9.0 ± 4.9	9.1 ± 5.2

Furthermore, multiple regression analysis revealed that forefoot height and digitus minimus angle were significant predictors of hallux angle displacement on the rotational side. The regression model yielded a coefficient of determination (R²) of 0.135 and an adjusted R2 of 0.115, with F (2, 87) = 6.803, P = 0.002. The standardized regression coefficients (β) were β = -0.281 (P = 0.007) for forefoot height and β = -0.202 (P = 0.048) for digitus minimus angle. The VIF was < 1.020, indicating minimal multicollinearity (Table 3). In summary, greater forefoot height and more varus displacement of the digitus minimus on the rotational side were associated with greater varus displacement of the hallux.

**Table 3:** Multiple regression analysis of factors influencing hallux angle on the rotational side Overall model statistic: R² = 0.135, Adj. R² = 0.115, F (2, 87) = 6.80, P < 0.002. The table displays the results of a multiple linear regression model examining predictors of hallux valgus angle on the rotational side. Negative β coefficients indicate that increases in the predictor variable are associated with a decrease in the hallux angle (i.e., varus displacement). Both forefoot height and digitus minimus angle were statistically significant predictors. VIF values < 1.5 indicate minimal multicollinearity. cm: centimeter; CI: confidence interval; VIF: Variance Inflation Factor.

Variables (Predictor)	β (non-standardized)	95% CI (Lower)	95% CI (Upper)	β (standardized)	P-value	VIF
Constants	1.533	0.694	2.373	-	0.000	-
Forefoot height (cm)	-0.628	-1.075	-0.180	-0.281	0.007	1.020
Digitus minimus angle (º)	-0.223	-0.445	-0.002	-0.202	0.048	1.020

In contrast, on the non-rotational side, navicular height, digitus minimus angle, forefoot height, and midfoot inclination angle were significant predictors of hallux angle displacement. The regression model yielded an R² of 0.408 and an adjusted R² of 0.380, with F (4, 85) = 14.629, P < 0.001. The standardized regression coefficients were: β = -0.418 (p < 0.001) for navicular height, β = -0.287 (P = 0.002) for digitus minimus angle, β = -0.250 (P = 0.002) for forefoot height, and β = -0.194 (P = 0.020) for midfoot inclination angle. The VIF values were all <1.579, suggesting minimal multicollinearity (Table 4). In summary, lower navicular height, greater varus displacement of the digitus minimus, lower forefoot height, and increased eversion of the midfoot inclination angle on the non-rotational side were associated with increased valgus displacement of the hallux.

**Table 4:** Multiple regression analysis of factors influencing hallux angle on the non-rotational side Overall model statistic: R² = 0.408, Adj. R² = 0.380, F (4, 85) = 14.63, P < 0.001. This table presents the results of a multiple regression analysis to identify foot shape parameters associated with hallux valgus angle on the non-rotational side. All listed predictors were statistically significant. Negative β coefficients indicate that higher values of these parameters are associated with greater valgus displacement of the hallux. The model demonstrated good explanatory power (Adjusted R² = 0.380), and VIF values indicated low multicollinearity among variables. cm: centimeter; CI: confidence interval; VIF: Variance Inflation Factor.

Variables (Predictor)	β (non-standardized)	95% CI (Lower)	95% CI (Upper)	β (standardized)	P-value	VIF
Constants	0.134	-0.390	0.659	-	0.612	-
Navicular height (cm)	-1.062	-1.491	-0.632	-0.516	0.000	1.579
Digitus minimus angle (º)	-0.245	-0.371	-0.118	-0.332	0.000	1.071
Forefoot height (cm)	-0.459	-0.743	-0.175	-0.276	0.002	1.057
Midfoot inclination angle (º)	-0.059	-0.108	-0.010	-0.244	0.020	1.515

## Discussion

In this study, we compared foot morphology under three conditions: in a standing posture, with the rotational side under load, and with the non-rotational side under load. These comparisons enabled identification of side-specific morphological adaptations and their distinct contributions to hallux valgus displacement. Changes in foot shape and hallux angle due to rotational motions are influenced by the kinetic chain generated by internal and external rotation of the lower leg [17]. In addition to this kinetic chain effect, foot shape is also influenced by the loading position, with lower and more everted foot indices observed under weight-bearing conditions [11]. Verbal instructions limited lateral weight shift; however, rotational motion still caused anterior-posterior load redistribution. On the rotational side, the load shifted toward the hindfoot; on the non-rotational side, it shifted toward the forefoot. Since the calcaneus ground contact plane is positioned laterally to the talofemoral joint plane, hindfoot loading generated eversion forces on the medial-lateral malleolus and calcaneal inclination angles [11]. However, on the rotational side, the kinetic chain associated with lower leg external rotation and subtalar inversion exerted inversion forces on the hindfoot. These opposing forces-eversion from hindfoot loading and inversion from rotational kinetics-appeared to offset each other, resulting in no net angular change. In contrast, on the non-rotational side, lower leg internal rotation and subtalar eversion, combined with forefoot loading, acted through a more flexible foot structure [19], with the load shifted to the medial foot, contributing to hallux valgus deformity via anteromedial loading and subtalar eversion.

Multiple regression analysis revealed that on the rotational side, hallux angle decreased with increased forefoot height and greater varus displacement of the digitus minimus. Conversely, on the non-rotational side, the hallux angle increased with lower navicular height, greater varus displacement of the digitus minimus, lower forefoot height, and increased eversion of the midfoot inclination angle. These findings suggest that forefoot and navicular height affect the length and tension of the transverse head of the adductor digitorum muscle. Prolonged external force on the foot due to internal lower leg rotation may disrupt muscle balance [23,24], contributing to the progression of hallux valgus. Among the influencing factors, navicular height was most significant: a lower navicular height corresponded to greater valgus displacement of the hallux, consistent with prior research [3]. Navicular height reflects the support of the medial longitudinal arch; therefore, its reduction can lead to first metatarsal abduction and eventual hallux valgus.

Moreover, hallux valgus is also linked to splay foot [25] and tailor’s bunion [26], aligning with our results. The mechanism behind tailor’s bunion remains unclear; however, it is thought that plantar friction [27] with the ball of the hallux and digitus minimus acting as fulcrums may cause angular deformation. Furthermore, calcaneus eversion is associated with reduced foot stiffness [19], which may lead to deformities in both the hallux and digitus minimus.

Based on these findings, adding support to the medial [28] or transverse forefoot [29] arch and distributing plantar pressure [30] may help reduce hallux valgus progression. In particular, transverse arch support could directly decrease the hallux valgus angle, while medial arch support may help prevent it. Notably, the forefoot, midfoot, and medial-lateral malleolus inclination angles changed in directions opposite to that of the calcaneus inclination angle, emphasizing the importance of considering their relative spatial alignment when creating the insole. However, the improvement in the hallux valgus angle on the rotation side showed a low R² value, suggesting that customization according to the subject is necessary.

A key limitation of this study is that only healthy adults were included, limiting the generalizability of the findings to individuals with osteoarthritis or foot disorders. Furthermore, load distribution during rotational motion may vary based on spinal alignment or sports history. Quantifying rotational motion is inherently difficult, and individual differences in body flexibility, foot stiffness, and calcaneus-lower leg kinetic chain ratios [17] may have influenced the results. The R² value in the regression model was relatively low, suggesting that some participants may have been at risk for hallux valgus. We aimed to capture subtle changes; however, it was impossible to achieve zero skin artifacts [13]. Furthermore, since the 3D foot scanner’s measurement points were treated as independent variables, it remains unclear which specific joint movements in the foot contributed to the observed displacement. Future studies examining the effects of insole use may provide further insight into the clinical application of these findings. A notable strength of this study is the use of a 3D foot scanner under both static and dynamic weight-bearing conditions, allowing for detailed evaluation of foot morphology changes. Furthermore, the simultaneous assessment of multiple angular and height-based indices enabled a comprehensive biomechanical analysis of hallux valgus dynamics during rotational loading.

## Conclusions

This study advances the understanding of how rotational motion affects foot morphology and hallux angles. Forefoot and navicular heights significantly influenced hallux angle displacement, underscoring the importance of foot morphology in hallux alignment. The forefoot, midfoot, and medial-lateral malleolus inclination angles demonstrated angular changes opposite to those of the calcaneus, indicating complex spatial dynamics within the foot. These findings enhance our understanding of the pathophysiology of hallux valgus and may inform the development of targeted insole-based therapies.
